# Do Social Norms for Cigarette Smoking and Nicotine Vaping Product Use Predict Trying Nicotine Vaping Products and Attempts to Quit Cigarette Smoking Amongst Adult Smokers? Findings From the 2016–2020 International Tobacco Control Four Country Smoking and Vaping Surveys

**DOI:** 10.1093/ntr/ntac212

**Published:** 2022-09-09

**Authors:** Hua-Hie Yong, Ruth Chow, Katherine East, James F Thrasher, Sara C Hitchman, Ron Borland, K Michael Cummings, Geoffrey T Fong

**Affiliations:** School of Psychology, Deakin University, Geelong, Victoria, Australia; School of Psychology, Deakin University, Geelong, Victoria, Australia; Department of Addictions, Institute of Psychiatry, Psychology & Neuroscience, King’s College London, London, UK; Department of Health Promotion, Education, and Behavior, Arnold School of Public Health, University of South Carolina, Columbia, SC, USA; Department of Communication and Media Research, University of Zurich, Zurich, Switzerland; Melbourne Centre for Behaviour Change, School of Psychological Sciences, University of Melbourne, Melbourne, Victoria, Australia; Department of Psychiatry & Behavioral Sciences, Medical University of South Carolina, Charleston, SC, USA; Department of Psychology, University of Waterloo, Waterloo, Ontario, Canada; Ontario Institute for Cancer Research, Toronto, Ontario, Canada

## Abstract

**Introduction:**

To examine whether perceived injunctive and descriptive social norms towards cigarette and nicotine vaping product (NVP) use predicted subsequent trying NVPs and attempts to quit cigarette smoking amongst current smokers and whether associations varied across countries.

**Aims and Methods:**

Three waves of longitudinal cohort data from the International Tobacco Control Four Country Smoking and Vaping Survey were collected between 2016 and 2020 from 2290 adult smokers in Canada, Australia, England, and the United States who had never used NVPs at baseline (either wave 1 or wave 2) and followed up at the subsequent wave (wave 2 or wave 3, respectively) were analyzed using Generalized Estimating Equations.

**Results:**

Of the injunctive and descriptive norm measures for smoking and NVP use, NVP initiation was only independently predicted by the injunctive interpersonal norm for NVP use, with perceived approval of NVP use by important others predicting higher odds of trying NVPs (AOR = 1.65, 95% CI = 1.20 to 2.27). This predictive effect was independent of baseline quit intention with no country variations found. By contrast, making cigarette smoking quit attempts were independently predicted by both injunctive and descriptive interpersonal norms with perceived disapproval of smoking by important others (AOR = 1.65, 95% CI = 1.38 to 1.99) and close friends using NVPs (AOR = 1.37, 95% CI = 1.04 to 1.79), both associated with higher odds of smoking quit attempts.

**Conclusions:**

Adult smokers who perceive NVP use as normative, either because such behavior is socially approved or common within their close social networks, appear more inclined to try NVPs or make smoking quit attempts than smokers who do not.

**Implications:**

Social norms can shape a person’s behavior and result in behavior change. This study shows that initiation of NVP use behavior among smokers can be reliably predicted by their perception of whether NVP use is acceptable to those important to them within their close social networks. Similarly, any attempts to stop cigarette smoking can be predicted by their perception of how acceptable cigarette smoking is among those who are important to them and whether any of their close friends use NVPs. Changing social norms towards cigarette smoking and NVP use could therefore be incorporated into smoking cessation interventions to help smokers to quit and/or switch to NVP use.

## Introduction

Social norms influence behavior change.^1^ The influence of social norms on behavior initiation and cessation, such as smoking and vaping, is central to the Theory of Planned Behavior (TPB).^2^ TPB asserts that an individual’s actions are influenced by psychosocial variables such as injunctive and descriptive social norms.^3,4^ Injunctive social norms are an individual’s perception of whether society approves of certain actions, whereas descriptive norms reflect their perceptions of how individuals typically act and behave.^3,5^

As per TPB, smoking uptake and cessation can be socially influenced, with people more likely to start smoking if other people within their social networks also smoke^6,7^ and more likely to quit smoking if people within their social networks also quit smoking.^7^ As the use of nicotine vaping products (NVPs and aka e-cigarettes) shares behavioral similarities to traditional cigarette smoking, the influence of social networks on NVP use behavior is likely similar. TPB would predict that individuals who believe that their peers both approve of, and use, NVPs, should be more likely to use NVPs themselves.^8^

Consistent with the findings on smoking initiation,^6,7^ NVP initiation among adults is influenced by exposure to NVP use behavior through one’s social networks.^9–11^ A qualitative study conducted in the United Kingdom found the majority of study participants, comprised of current and former smokers, reported initially hearing about NVPs from family or friends who used NVPs, and were encouraged to start NVP use as a way to stop smoking.^9^ A recent systematic review further supported the strong influence of close social connections on NVP use, finding evidence to suggest that social factors, including social norms, play an important role in shaping NVP use behaviors, including initiation.^11^ The review also suggested cross-product social influences, indicated by studies showing that young people were more likely to start NVP use if their friends and family members smoked.^11^

A recent study in England also identified that smokers’ quitting motivation and behavior were positively influenced by exposure to NVP use by others.^10^ Jackson et al.^10^ found that smokers with regular exposure to NVP use by others were more motivated to quit smoking and more likely to have engaged in quit attempts in the past 12 months in cross-sectional analyses, but not in the past 6 months in longitudinal analyses. Conversely, those regularly exposed to cigarette smoking by others were less motivated to quit smoking. These findings suggest that reference groups with greater proximity to an individual, such as their close friends and family members, are highly influential in shaping behavior.^12,13^ Given the dearth of prospective studies examining the relative influence of varying social norms towards NVP use and cigarette smoking on NVP use behavior, and the possibility for differences, more research of this kind is warranted.

Whilst proximal social influences within social networks affect one’s behavior, norms at the societal level, that represent the broader environment, also play a role.^11^ This is consistent with a socio-ecological model of behavior influences which posits that a person’s behavior is influenced by factors at multiple levels from intrapersonal factors at the lowest level to external factors from the immediate social environment and ultimately to those factors at the societal or country level representing the much broader physical and policy environment one resides in.^14^ Recent studies have found that the degree of regulation restrictiveness across countries is correlated with the social acceptability of NVPs.^15,16^ Due to ongoing debate regarding the safety of NVPs, there are wide variations in the restrictiveness of NVP regulations across countries.^17^ In 2018, the retail sale of vaping products with and without nicotine has been banned in 27 countries.^17^ In particular, Australia has very restrictive NVP regulations and prohibits the retail sale of NVPs unless prescribed as a smoking cessation aid.^18^ Conversely, Canada, England, and the United States have a more relaxed approach to NVP regulation, with the retail sale of NVPs permissible to adults.^17^ Smokers in England, with comparatively less restrictive NVP policies than their Australian counterparts, have been found to be much more likely to view NVP use as socially acceptable.^15,16^ This suggests that greater exposure to public NVP use behaviors and more relaxed regulations may be associated with the development of greater positive injunctive norms regarding NVP use.^15,16^ However, when Wadsworth et al.^19^ examined smokers’ exposure to NVP advertising and perceptions of NVPs in varying NVP regulatory environments, they found minimal country differences in positive attitudes towards NVP use. The aforementioned discrepancies in literature emphasize the importance of examining how differences in the restrictiveness of country regulations may impact normative beliefs towards NVP use.

A longitudinal study by McDermott et al.^20^ used data from the International Tobacco Control (ITC) project conducted in seven European countries to examine the influence of descriptive and injunctive norms on NVP uptake amongst current and former smokers. They found that measures of descriptive norms, including having at least one close friend that used NVPs and frequency of seeing NVP use in public places, were positively associated with NVP initiation, whereas the number of five closest friends who smoked showed negative associations. However, NVP initiation was not influenced by injunctive norms towards NVP use or cigarette smoking. Once the other norm variables and sociodemographics were adjusted for, the reported frequency of seeing NVP use in public was the only remaining significant association with NVP initiation.^20^

Consistent with our past research on social norms for cigarette smoking (eg Hosking et al.^21^), McDermott et al.^20^ also found some measures of descriptive and injunctive social norms for NVP use predicted intention to quit cigarette smoking. With one exception, none of these measures predicted making an attempt to subsequently quit smoking. Smokers who reported having three or more close friends who smoke cigarettes were less likely to have made a subsequent quit attempt compared to those with fewer smoking friends. Given the lack of research on the influence of descriptive and injunctive norms on NVP uptake and smoking cessation attempts, the findings from McDermott et al.^20^ deserve replication. Moreover, it is unclear whether their findings are generalizable to countries outside of Europe with different NVP regulations.

Understanding the influence of social norms on NVP use and smoking quit attempts has important implications for practice and policy. Within clinical contexts, the findings could inform interventions that target social factors to support or deter NVP use and also to more effectively promote cigarette smoking cessation.^22^ At the policy level, the findings could confirm or help refine models/frameworks that place social norms on the pathway between policies and changes in cigarette smoking/NVP use prevalence.^23,24^

Thus, this study aimed to prospectively examine (1) whether perceived injunctive and descriptive norms for cigarette smoking and NVP use among adult smokers who had never used NVPs were predictive of any subsequent trial of NVPs and attempts to quit cigarette smoking, and (2) whether the predictive relationships with NVP ever use varied by country given the different regulatory environments for NVPs.

## Methods

### Participants and Design

Longitudinal data from adult participants aged 18 years and older in Canada, Australia, England, and the United States who participated in wave 1 (collected from July to November 2016), wave 2 (February to July 2018), and wave 3 (February to June 2020) of the ITC Four Country Smoking and Vaping Survey were analyzed. Participants were sampled from the following cohorts: (1) Recontact current or former smokers from the original ITC survey, (2) Current or former smokers replenished from country-specific panels, and (3) Newly recruited NVP users from country-specific panels. To replenish those lost to attrition, new participants were recruited in each follow-up survey wave according to wave 1 sampling criteria.

To meet the inclusion criteria for the current study, the analytic sample only consisted of those who provided data for at least one wave-to-wave transition across the three waves, defined as current (daily, weekly, or monthly) smokers who had never used NVPs at their baseline wave (either wave 1 or 2) and provided outcome data the following wave (wave 2 or 3, respectively). This resulted in a final sample of 2290 individuals providing 3015 observations across two baseline-to-follow-up wave pairs in total (68.3% providing data for one baseline-to-follow-up wave pair and 31.7% for two baseline-to-follow-up wave pairs).

### Measures

#### Social Norm Variables (Predictors) Assessed at Baseline (Wave 1 or 2)

Questions on norms for smoking and NVP use were assessed at each baseline survey wave. For each product type, injunctive norms were assessed at both the proximal interpersonal level (ie important people’s approval of smoking or vaping) and distal societal level (ie perceptions of the general public’s attitudes towards smoking and towards vaping). Descriptive norms were only assessed at the proximal interpersonal level by first asking, “How many friends or acquaintances do you spend time with on a regular basis?”, with response options between zero and five. If participants reported at least one friend or acquaintance, they were asked about their number of close friends who smoked or used NVPs. Details of specific survey questions on both injunctive and descriptive norms and response options are outlined in Supplementary Table S1. This set of social norm variables has been found to be generally well understood by adults in cognitive testing^25^ and also have been successfully used in our past research.^20,26^ Descriptive norms at the distal societal level were not assessed in the survey.

#### NVP Ever Use (Outcome) Assessed at Follow-Up (Wave 2 or 3)

Respondents were asked, “Have you ever used an e-cigarette or vaping device, even one time?” Responses included: Yes, no, or I have never heard of e-cigarettes/vaping devices. Responses were recoded into “yes” versus “other” (all else combined) for the purpose of analysis.

#### Control Variables Assessed at Baseline (Wave 1 or 2)

Control variables, all assessed at baseline, included country of residence (Canada, Australia, England, the United States), gender (male, female), ethnicity (white, non-White), age group (18–24, 25–39, 40–54, ≥55 years), highest level of education (low [<high school], moderate, high [≥completed university]), and annual household family income (low [<$30 000 in Canada, United States, Australia; <£15 000 in England], moderate, high [≥$60 000 in Canada, United States, Australia; >£50 000 in England], no information).^27^ Baseline smoking-related variables were smoking frequency (daily, weekly, and monthly) and quit intention (no plan, beyond 6 months, next 6 months, next month, refused, or don’t know). Methodological variables included the wave recruited into the study and the survey wave.

### Data Analysis

Simple cross-tabulations were used to examine the sample characteristics of participants and determine rates of NVP ever use and cigarette smoking quit attempts at follow-up by baseline predictor variables. Weighted prevalence estimates of injunctive and descriptive norms for cigarette smoking and NVP use were also computed.

Generalized Estimating Equations (GEE) logistic regression models were used to examine the predictive relationship between the set of social norm variables and behavioral outcomes of interest (ie NVP ever use or made attempts to quit cigarette smoking by follow-up wave). GEE can account for the correlated nature of the data and maximize the statistical power of the sample through the estimation of responses across different waves. As the outcome variables were binary, parameter estimates were obtained using the logit link function with robust variance and an unstructured correlation structure for the working correlation matrix.

GEE modeling was conducted in stages, involving *individual* and *concurrent* analyses for predicting both outcomes. For both outcomes, modeling began with *individual* analysis by examining the relationship between each baseline social norm predictor and follow-up survey behavioral outcomes adjusting for potential sociodemographic and methodological confounders such as gender, ethnicity, age, income, education, country, wave recruited into study, and survey wave (model 1). To determine the independent effects of the social norm variables, we conducted *concurrent* analyses. Firstly, the four injunctive norm variables adjusting for sociodemographic confounders were entered into the model (model 2), then the two interpersonal level descriptive norm variables were added (model 3). To determine if the effects of these social norm variables were independent of smoking quit intentions, we added quit intentions into the model (model 4). To predict making smoking quit attempts, NVP ever uses reported at follow-up were added to the model as a final step to determine if social norm effects were independent of any subsequent NVP use (model 5).

Given the very different regulatory environments for NVPs across the four studied countries, the second study aim was to explore for any country differences in the above predictive relationships with NVP ever use. This was done by adding norm-by-country interaction terms into the model.

For ease of interpretation, odds ratio [OR] and 95% confidence intervals [CI] were estimated and reported. All analyses were conducted using Stata version 17. Tests of association were two-tailed (alpha < 0.05).

## Results

### Sample Characteristics and Social Norm Prevalence Estimates

Based on cross-tabulation results, baseline sample characteristics of never vapers and prevalence estimates of injunctive and descriptive norms are displayed in Tables 1 and 2, respectively. With the exception of Canada, all other countries had a greater percentage of male participants (see Table 1). The majority of participants were of White ethnicity with low to moderate education levels. The ≥55 years age category had the largest proportion of participants.

**Table 1.** Baseline Sample Characteristics

Variables	Canada (CA) (%) *N* = 657	Australia (AU) (%) *N* = 539	England (EN) (%) *N* = 668	United States (US) (%) *N* = 426	Total (%) *N* = 2290
Gender					
Male	42.62	51.76	54.49	52.58	50.09
Female	57.38	48.24	45.51	47.42	49.91
Ethnicity					
White	88.89	89.61	94.76	78.64	88.86
Non-White	9.44	10.39	3.89	20.89	10.17
Ref/DK	1.67	0.00	1.35	0.47	0.96
Age group (years)					
18–24	4.57	0.74	3.59	1.88	2.88
25–39	13.24	11.69	12.87	14.08	12.93
40–54	37.60	37.29	37.72	21.36	34.54
≥55	44.60	50.28	45.81	62.68	49.65
Education					
Low	33.18	36.55	41.32	42.72	38.12
Mod	42.77	40.07	33.53	37.09	38.38
High	23.44	23.19	23.35	20.19	22.75
Ref/DK	0.61	0.19	1.80	0.00	0.74
Income					
Low	36.23	37.11	25.15	36.62	33.28
Mod	25.42	21.15	46.86	34.27	32.31
High	31.05	34.88	18.86	28.40	27.90
Ref/DK	7.31	6.86	9.13	0.70	6.51

Ref = refused; DK = don’t know; N = total unique individuals; percentages are unweighted.

**Table 2.** Weighted Prevalence Estimates of Descriptive and Injunctive Norms for Cigarette Smoking and Nicotine Vaping Product Use of Baseline Sample, by Country

Social norm variables	Canada (%) *N* = 852	Australia (%) *N* = 725	England (%) *N* = 862	US (%) *N* = 576	Total *N* = 3015
**Injunctive societal norms**					
Public’s attitudes towards cigarette smoking					
Approves	3.0	3.9	1.9	6.3	3.6
Neither	8.5	13.9	15.2	11.1	12.1
Disapproves	85.7	77.9	78.5	73.4	79.5
Refused/DK	2.7	4.2	4.3	9.2	4.8
Public’s attitudes towards NVP use					
Approves	15.4	9.0	19.3	11.0	14.1
Neither	18.1	19.0	29.2	16.5	21.0
Disapproves	41.0	27.1	27.0	35.5	32.9
Refused/DK/NHV	25.5	44.9	24.6	37.0	32.1
**Injunctive interpersonal norms**					
Important people’s thoughts on your cigarette smoking					
Most/all approve	6.7	5.6	6.3	7.7	6.5
Half approve & half disapprove	23.0	29.1	27.6	22.6	25.6
Most/all disapprove	62.3	56.0	51.2	51.0	55.5
Refused/DK	8.0	9.4	15.0	18.7	12.4
Important people’s thoughts on your NVP use					
Most/all approve	11.6	8.4	13.6	9.7	11.0
Half approve & half disapprove	18.4	16.3	23.7	12.2	18.1
Most/all disapprove	25.6	19.3	16.2	24.2	21.3
Refused/DK/NHV	44.3	55.9	46.6	53.9	49.6
**Descriptive interpersonal norms**					
Number of 5 closest friends smoking cigarettes					
0	25.8	21.4	21.4	20.5	22.5
1–5	56.7	56.2	52.7	48.3	53.8
Refused/DK/NRF	17.6	22.3	25.9	31.2	23.7
Number of 5 closest friends using NVPs					
0	71.1	71.9	59.0	57.7	65.2
1-5	7.6	4.2	13.8	6.2	8.2
Refused/DK/NRF	21.4	23.9	27.2	36.1	26.6

*N* = number of observations; NHV = never heard of NVPs; NRF = not regular friends.

As seen in Table 2, the majority of participants from all countries believed that the public disapproves of smoking, with Canadian participants indicating the strongest disapproval. England had the highest percentage of participants who believed that the public approves of NVP use (19.3%) followed by Canada (15.4%), the United States (11.0%), and Australia (9.0%). Canada had the most participants who reported no cigarette smoking friends among their five closest friends (25.8%) and the United States the least (20.5%). England had the most participants with at least one friend using NVPs (13.8%) and Australia the least (4.2%).

### Prospective Associations Between Social Norms and Having Tried NVPs

Table 3 presents the GEE results from the *individual* (model 1) and *concurrent* analyses (models 2–4).

**Table 3.** Generalized Estimating Equation Analysis Predicting Trying Nicotine Vaping Products Between Waves (*n* = 2290; *N* = 3015)

Baseline predictor variables	% Trying vaping between waves	Model 1	Model 2	Model 3	Model 4
		AOR [95% CI]	AOR [95% CI]	AOR [95% CI]	AOR [95% CI]
**Injunctive societal norms**					
Public’s attitude towards cigarette smoking					
Approves	43.1	2.04 [1.16 to 3.56]**	1.48 [0.81 to 2.72]	1.43 [0.78 to 2.61]	1.37 [0.74 to 2.53]
Neither	23.5	*ref*	*ref*	*ref*	*ref*
Disapproves	20.8	1.02 [0.77 to 1.37]	1.00 [0.74 to 1.36]	0.99 [0.73 to 1.35]	0.97 [0.72 to 1.32]
Refused/DK	20.7	0.89 [0.51 to 1.56]	1.13 [0.64 to 2.00]	1.13 [0.64 to 2.00]	1.10 [0.62 to 1.95]
Public’s attitude towards NVP use					
Approves	32.1	1.37 [1.02 to 1.86]*	1.05 [0.75 to 1.46]	1.05 [0.76 to 1.47]	1.05 [0.75 to 1.46]
Neither	25.0	*ref*	*ref*	*ref*	*ref*
Disapproves	19.3	0.84 [0.65 to 10.8]	0.97 [0.74 to 1.28]	0.97 [0.74 to 1.28]	0.97 [0.73 to 1.28]
Refused/DK/NHV	17.8	0.81 [0.62 to 1.05]	1.04 [0.77 to 1.40]	1.03 [0.76 to 1.39]	1.03 [0.76 to 1.40]
**Injunctive interpersonal norms**					
Important people’s thoughts on your cigarette smoking					
Most/All approve	26.8	1.11 [0.75 to 1.64]	0.88 [0.57 to 1.36]	0.89 [0.58 to 1.38]	0.93 [0.60 to 1.44]
Half approve & half disapprove	22.8	*ref*	*ref*	*ref*	*ref*
Most/All disapprove	21.7	1.01 [0.82 to 1.26]	1.11 [0.88 to 1.39]	1.11 [0.88 to 1.39]	1.05 [0.83 to 1.33]
Refused/DK	15.4	0.61 [1.14 to 4.19]**	0.72 [0.51 to 1.03]	0.72 [0.50 to 1.03]	0.74 [0.52 to 1.05]
Important people’s thoughts on your NVP use					
Most/All approve	36.7	1.65 [1.20 to 2.27]**	1.61 [1.14 to 2.27]**	1.60 [1.13 to 2.27]**	1.56 [1.10 to 2.22]**
Half approve & half disapprove	28.3	*ref*	*ref*	*ref*	*ref*
Most/All disapprove	19.6	0.69 [0.51 to 0.93]*	0.69 [0.50 to 0.96]*	0.70 [0.50 to 0.97]*	0.70 [0.51 to 0.97]*
Refused/DK/NHV	17.2	0.66 [0.52 to 0.85]***	0.69 [0.52 to 0.92]*	0.70 [0.52 to 0.93]*	0.70 [0.53 to 0.94]*
**Descriptive interpersonal norms**					
Number of 5 closest friends smoking cigarettes					
0	20.4	*ref*	NA	*ref*	*ref*
1–5	22.0	1.00 [0.80 to 1.24]	NA	0.95 [0.76 to 1.20]	0.96 [0.76 to 1.21]
Refused/DK/NRF	21.7	0.97 [0.73 to 1.27]	NA	0.91 [0.52 to 1.57]	0.95 [0.55 to 1.64]
Number of 5 closest friends using NVPs					
0	20.5	*ref*	NA	*ref*	*ref*
1–5	28.2	1.38 [1.00 to 1.89]*	NA	1.25 [0.90 to 1.73]	1.24 [0.89 to 1.72]
Refused/DK/NRF	22.1	1.02 [0.81 to 1.27]	NA	1.12 [0.68 to 1.84]	1.10 [0.67 to 1.80]
**Quit intention**					
No plans	16.0	NA	NA	NA	*ref*
Beyond 6 months	26.3	NA	NA	NA	1.67 [1.28 to 2.18]***
Next 6 months	22.5	NA	NA	NA	1.38 [1.02 to 1.85]*
Next month	24.0	NA	NA	NA	1.47 [1.01 to 2.14]*
Refused/DK	19.8	NA	NA	NA	1.33 [0.96 to 1.85]

*n* = number of unique individuals; *N* = number of observations; AOR = adjusted odds ratio; CI = confidence interval; *ref* = reference group; NHV = never heard of NVPs; NRF = no regular friends; NA = not applicable; statistically significant AORs are indicated in bold.

Model 1 = individual analysis of social norm measures for smoking and NVP use plus covariates such as country, gender, ethnicity, age, education, income, smoking status, wave of recruitment, and survey wave.

Model 2 = concurrent analysis of norm measures after adding in injunctive norm measures for smoking and NVP use plus covariates such as country, gender, ethnicity, age, education, income, smoking status, wave of recruitment, and survey wave.

Model 3 = concurrent analysis of norm measures after adding in descriptive norm measures for smoking and NVP use plus covariates such as country, gender, ethnicity, age, education, income, smoking status, wave of recruitment, and survey wave.

Model 4 = concurrent analysis of full set of social norm measures for smoking and NVP use after adding in quit intention plus covariates such as country, gender, ethnicity, age, education, income, smoking status, quit intention, wave of recruitment, and survey wave.

#### Injunctive Norms

Of the four injunctive norm measures, the injunctive interpersonal norm around NVP use was the only measure showing an independent association with subsequent NVP ever use (model 2). Baseline smokers who believed that all or nearly all of the important people in their lives approved of their NVP use were more likely to have tried NVPs (adjusted odds ratio [AOR] = 1.61, 95% confidence interval [CI] = 1.14 to 2.27, *p* = .007). Those who believed important people disapproved of their NVP use (AOR = 0.69, 95% CI = 0.50 to 0.96, *p* = .027) and those who refused to answer or expressed “don’t know”, were less likely (AOR = 0.69, 95% CI = 0.52 to 0.92, *p* = .012) to have tried NVPs by the follow-up wave than those who believed that half of the important people approved and half disapproved of their NVP use. These predictive effects remained essentially unchanged after adjusting for the two descriptive interpersonal norms around cigarette smoking and NVP use (model 3) and for quit intention (model 4) which had a positive association with NVP ever use. Injunctive interpersonal norm towards smoking was not independently associated with having tried NVPs, except for a subsample of those who refused to answer or expressed “don’t know” who were less likely to try NVPs in the *individual* analyses (AOR = 0.61, 95% CI = 0.43 to 0.87, *p* = .006). Both measures of injunctive societal norms, one towards cigarette smoking and the other towards NVP use, were significantly associated with trying NVPs in the follow-up survey in *individual* analyses with baseline smokers who believed that the general public approved of smoking (AOR = 2.04, 95% CI = 1.16 to 3.56, *p* = .012) and NVP use, respectively (AOR = 1.37, 95% CI = 1.02 to 1.86, *p* = .036), being more likely to have tried NVPs by follow-up survey.

#### Descriptive Interpersonal Norms

*Individual* analyses (model 1) indicated that of the two descriptive norms, only the number of close friends using NVPs was significantly associated with subsequently trying NVPs. Those who reported at least one close friend who used NVPs were more likely to have tried NVPs (AOR = 1.38, 95% CI = 1.00 to 1.89, *p* = .049) but this effect was no longer significant after adjusting for other norm measures in *concurrent* analyses (model 3).

No significant social norm by country interactions was found for any of the associations between baseline social norm measures and subsequent NVP ever use.

### Prospective Associations Between Social Norms and Cigarette Smoking Quit Attempts

Supplementary Table S2 presents the results from the GEE analyses predicting cigarette smoking quit attempts.

#### Injunctive Norms

Of the four measures of injunctive norms, only the interpersonal norm relating to cigarette smoking was significantly and independently associated with subsequent smoking quit attempts (model 2). Baseline smokers who believed that all or nearly all of the important people in their lives disapprove of their smoking were more likely to make smoking quit attempts (AOR = 1.56, 95% CI = 1.29 to 1.89, *p* < .001). Injunctive societal norm towards NVP use was not related to smoking quit attempts in both *individual* and *concurrent* analyses. Injunctive interpersonal norm towards NVP use was only significantly related to cigarette smoking quit attempts in *individual* analyses whereby those who believed that all or nearly all of the important people in their lives disapprove of their NVP use were more likely to have made cigarette smoking quit attempts (AOR = 1.31, 95% CI = 1.02 to 1.67, *p* = .032).

#### Descriptive Interpersonal Norms

Of the two descriptive interpersonal norm measures, only the norm towards NVP use was significantly related to making cigarette smoking quit attempts (model 3). Compared to those with no close friends who used NVPs, smokers who reported at least one close friend using NVPs were more likely to make subsequent smoking quit attempts (AOR = 1.39, 95% CI = 1.05 to 1.84, *p* = .021) but those who responded “don’t know/refused to answer/no regular friends” were less likely to do so (AOR = 0.60, 95% CI = 0.38 to 0.93, *p* = .022). After controlling for baseline quit intention (model 4), this predictive effect remained significant and persisted even after adjusting for any NVP use by a follow-up survey (model 5). Baseline quit intention and follow-up NVP ever use were both positively associated with making cigarette smoking quit attempts.

## Discussion

This study found injunctive societal norms towards cigarette smoking or NVP use, injunctive interpersonal norms around NVP use, and descriptive interpersonal norms around NVP use were all predictive of subsequently trying NVPs among baseline smokers who had never used NVPs. However, only the injunctive interpersonal norm around NVP use (ie perceived approval of NVP use from important people) showed effects independent of other norm measures and baseline quit intention. Our analyses also revealed that injunctive societal norms towards smoking and injunctive interpersonal norms for both smoking and NVP use were predictive of attempts to quit smoking. Interestingly, descriptive interpersonal norms towards smoking had an opposite effect to that of NVP use: smokers had a reduced likelihood of making smoking quit attempts if they had at least one close friend smoking, but an increased likelihood of making smoking quit attempts if they had at least one friend who used NVPs. Of note is that the predictive effect of the injunctive interpersonal NVP norm measure on quit attempts was independent of quit intention and any subsequent NVP behavior, suggesting that it may have a direct influence on smoking cessation.

The finding that perceived societal approval of NVP use predicting trying NVPs contradicts that of McDermott et al.^20^—the difference in findings is likely due to the much lower NVP use rate at the time of their study. However, our study is consistent with other studies finding that perceived social acceptability of NVP use promotes NVP initiation,^11,28^ particularly if such behavior is perceived to be endorsed by those within their close social network. Nevertheless, this finding needs further replication as the effect was not independent of other norm measures and it is unclear whether this was due to confounding or insufficient statistical power.

Our study did not find clear evidence of the influence of cigarette smoking norms on trying NVPs among current smokers. This suggests that normative beliefs about smoking, whether descriptive, or injunctive, do not appear to affect whether a smoker commences NVP use or not. This may be because smokers can clearly recognize the distinct differences between the two behaviors and therefore, their perception of smoking-related norms should not affect their own beliefs and actions regarding to NVP use.^13^ If confirmed, this finding implies that any interventions/campaigns aiming to change norms towards smoking may not impact NVP use. Nevertheless, given the relatively strong skew towards perceived social unacceptability of cigarette smoking, future research on injunctive norms may require the use of more sensitive measures.

Consistent with past research,^21,26^ our results suggest that an anti-smoking social context may motivate and facilitate smoking cessation among smokers, however, the influence of the proximal social context is stronger and more direct than the broader societal level influence. Unlike Jackson et al.^10^ and McDermott et al.^20^ who found no independent associations between social norms for NVP use or cigarette smoking and cigarette smoking quit attempts, our study found that injunctive norms for cigarette smoking and descriptive norms for NVP use at the interpersonal level had predictive effects on smoking quit attempts. The effect of cigarette smoking injunctive norm on smoking quit attempts appears to be mediated through smoking quit intentions whereas the effect of NVP use descriptive norm was independent of smoking quit intentions and subsequent NVP use, thus suggesting a more direct effect. The different findings from past studies may be due to methodological and/or sampling differences between studies. For example, Jackson et al.^10^ asked about exposure to NVP use by a range of people (from home, work, or other places they visited regularly) whereas McDermott et al.^20^ and our study assessed NVP use among close friends. Nevertheless, our findings suggest that a pro-NVP use culture within one’s close social network may stimulate smokers to try to quit smoking, regardless of their initial smoking-related quitting motivation or subsequent NVP use behaviors. Thus, our findings imply that policies designed to promote a pro-NVP use culture are unlikely to undermine smoking cessation among current smokers and may have the added benefit of changing the minds of some smokers with no plans to quit smoking to attempt to quit. Further research could systematically explore whether the effects of a pro-NVP use social context on smokers’ behavior change are mediated through their motivation to quit smoking and/or commencing NVP use as a way to help them quit cigarette smoking.

The lack of any country differences in the predictive effect of social norms on any trial of NVPs suggests that their influences on NVP use behavior, both at the broader societal and proximal social network level, may be independent of country regulatory environments. This is despite evidence of regulatory environment affecting norms towards NVP use,^15^ the extent of exposure to NVP marketing,^19^ and the extent of NVP uptake.^16^

This study has several limitations. Firstly, the lack of variability in number of vaping friends (ie few participants reporting more than one friend using NVPs) precludes our ability to examine the influence of a greater number of NVP using friends on NVP ever use. This may have also contributed to the overall nonsignificant effect. Secondly, the use of a single-item norm measure assumes construct uni-dimensionality and may not adequately capture all aspects of the construct of interest; indeed, multi-item measures of injunctive social norms (eg “My friends/best friend/family/people important to me think I should/should not smoke”) have been found to be stronger predictors of intention to engage in behavior than single-item measures (eg “People important to me think I should/should not smoke”).^29^ Thirdly, this study did not examine how often or how much one’s friends smoke/use NVPs. This may affect how salient norms are which may subsequently influence the perceived acceptability of smoking/NVP use.^5,16^ Finally, social influences on NVP initiation could be due to the influence of homophily. Individuals tend to associate with others who hold similar beliefs and engage in similar behaviors.^30^ Consequently, they may start NVP use, not because of the influence of their friends who use NVPs but instead, due to their positive attitudes towards NVP use, they seek out friends that reinforce those beliefs and behaviors.^13^ Future studies could explore the bidirectional relationship between the number of friends who use NVPs and individual attitudes towards NVP use, in order to understand how they influence NVP use.

Despite these limitations, the study strengths included a longitudinal cohort design, a large demographically diverse sample, and four countries with divergent regulatory environments for comparison.

In conclusion, the research suggests that current smokers who are exposed to a pro-NVP use culture, both at a proximal social network level and also at a distal societal level, are likely to try NVPs or attempt to quit cigarette smoking. These social factors appear to be similarly influential across four high-income countries, despite varying NVP policies and regulations. These findings suggest that social norms are an important consideration when designing policies and clinical interventions relating to NVP use and smoking cessation. For example, policymakers may want to carefully consider how extending smoke-free policies to NVP use might affect the social acceptability of NVP use as this might have the unintended consequence of promoting misperceptions that NVPs are just as harmful as cigarette smoking and may discourage their use by current smokers wanting to quit smoking. Clinicians may also want to carefully consider the social context of their clients when recommending NVP use as an aid to quit smoking to make sure that there are no social barriers to NVP use. Focusing on the influence of social norms can provide greater clarity into how social factors affect behavior change. Further research is required to confirm whether the results of the current study can be replicated across similar and different sociocultural contexts.

## Supplementary Material

A Contributorship Form detailing each author’s specific involvement with this content, as well as any supplementary data, are available online at https://academic.oup.com/ntr.

ntac212_suppl_Supplementary_Tables

ntac212_suppl_Supplementary_Taxonomy-form

## Data Availability

In each country participating in the international Tobacco Control Policy Evaluation (ITC) Project, the data are jointly owned by the lead researcher(s) in that country and the ITC Project at the University of Waterloo. Data from the ITC Project are available to approved researchers 2 years after the date of issuance of cleaned data sets by the ITC Data Management Centre. Researchers interested in using ITC data are required to apply for approval by submitting an International Tobacco Control Data Repository (ITCDR) request application and subsequently to sign an ITCDR Data Usage Agreement. The criteria for data usage approval and the contents of the Data Usage Agreement are described online (http://www.itcproject.org).
