# Supplementary material for: Do Social Norms for Cigarette Smoking and Nicotine Vaping Product Use Predict Trying Nicotine Vaping Products and Attempts to Quit Cigarette Smoking Amongst Adult Smokers? Findings From the 2016–2020 International Tobacco Control Four Country Smoking and Vaping Surveys
Source: Nicotine Tob Res. 2022 Sep 9;25(3):505–13. doi: 10.1093/ntr/ntac212 (PMC9910125; doi:10.1093/ntr/ntac212)
Supplement: ntac212_suppl_Supplementary_Tables [file ntac212_suppl_supplementary_tables.docx]

**Table S1.** *Survey questions and response options of predictor variables*

| Predictor variable | Survey question | Response options |
| --- | --- | --- |
| **Injunctive societal norms** |  |  |
| Public attitudes towards cigarette smoking | What do you think the general public’s attitude is towards smoking cigarettes? | Strongly approves, somewhat approves, neither approves nor disapproves, somewhat disapproves, strongly disapproves, refused, don’t know (DK) |
| Public attitudes towards NVP use | What do you think the general public’s attitude is towards vaping / using e-cigarettes? | Strongly approves, somewhat approves, neither approves nor disapproves, somewhat disapproves, strongly disapproves, refused, DK |
| **Injunctive interpersonal norms** |  |  |
| Important people’s thoughts on your cigarette smoking | What do (would) people who are important to you think about you smoking cigarettes? | All or nearly all approve, most approve, about half approve and half disapprove, most disapprove, all or nearly all disapprove, refused, DK |
| Important people’s thoughts on your NVP use | What do (would) people who are important to you think about you using e-cigarettes/vaping devices? | All or nearly all approve, most approve, about half approve and half disapprove, most disapprove, all or nearly all disapprove, refused, DK |
| **Descriptive interpersonal norms** |  |  |
| Number of 5 closest friends smoking cigarettes | Of these 5 closest friends or acquaintances that you spend time with on a regular basis, how many of friends/acquaintances smoke ordinary cigarettes? | 0, 1, 2, 3, 4, 5, refused, DK |
| Number of 5 closest friends using NVPs | Of these 5 closest friends or acquaintances that you spend time with on a regular basis, how many of friends/acquaintances use e-cigarettes/vaping devices? | 0, 1, 2, 3, 4, 5, refused, DK |

**Table S2.** *Generalised Estimating Equation analysis predicting making smoking quit attempts between waves (n=2,290; N=3,015)*

| Predictor variables | % made quit attempts between waves | Model 1 |  |  | Model 2 |  | | Model 3 |  | | Model 4 | | |  | | Model 5 | | |
| --- | --- | --- | --- | --- | --- | --- | --- | --- | --- | --- | --- | --- | --- | --- | --- | --- | --- | --- |
|  |  | AOR [95% CI] |  |  | AOR [95% CI] |  | | AOR [95% CI] |  | | AOR [95% CI] | | |  | | AOR [95% CI] | | |
| **Injunctive societal norms** | |  |  |  |  |  | |  |  | |  | | |  | |  | | |
| Public's attitude towards smoking |  |  |  |  |  |  | |  |  | |  | | |  | |  | | |
| Approves | 36.9 | 1.04 [0.59-1.81] |  |  | 1.11 [0.62-2.02] |  | | 1.09 [0.60-1.96] |  | | 0.82 [0.38-1.74] | | |  | | 0.74 [0.34-1.61] | | |
| Neither | 28.9 | *ref* |  |  | *ref* |  | | *ref* |  | | *ref* | | |  | | *ref* | | |
| Disapproves | 39.4 | **1.46 [1.13-1.89]**** | | 1.31 [0.99-1.72] | | 1.30 [0.99-1.70] | | |  | | 1.11 [0.83-1.49] | 1.12 [0.82-1.51] | | | | |  |  |
| Refused/DK | 33.3 | 1.31 [0.84-2.05] |  |  | 1.47 [0.92-2.34] |  | | 1.46 [0.92-2.33] |  | | 1.28 [0.76-2.15] | | |  | | 1.27 [0.74-2.18] | | |
| Public's attitude towards NVP use |  |  |  |  |  |  | |  |  | |  | | |  | |  | | |
| Approves | 37.1 | 0.93 [0.71-1.21] |  |  | 0.95 [0.72-1.27] |  | | 0.95 [0.71-1.27] |  | | 0.94 [0.68-1.29] | | |  | | 0.94 [0.68-1.31] | | |
| Neither | 36.0 | *ref* |  |  | *ref* |  | | *ref* |  | |  | | |  | | *ref* | | |
| Disapproves | 41.8 | 1.07 [0.86-1.31] |  |  | 0.92 [0.73-1.16] |  | | 0.91 [0.73-1.14] |  | | 0.91 [0.70-1.17] | | |  | | 0.92 [0.71-1.19] | | |
| Refused/DK/NHV | 35.8 | 0.87 [0.71-1.08] |  |  | 0.89 [0.70-1.12] |  | | 0.91 [0.72-1.16] |  | | 0.94 [0.72-1.23] | | |  | | 0.94 [0.71-1.24] | | |
| **Injunctive interpersonal norms** | |  |  |  |  |  | |  |  | |  | | |  | |  | | |
| Important people's thoughts on your smoking |  |  |  |  |  |  | |  |  | |  | | |  | |  | | |
| Most/All approve | 31.6 | .96 [0.67-1.38] |  |  | 1.01 [0.70-1.46] |  | | 1.03 [0.71-1.49] |  | | 1.11 [0.72-1.71] | | |  | | 1.13 [0.72-1.77] | | |
| Half approve & half disapprove | 31.4 | *ref* |  |  | *ref* |  | | *ref* |  | | *ref* | | |  | | *ref* | | |
| Most/All disapprove | 43.7 | **1.65 [1.38-1.99]***** | |  | **1.56 [1.29-1.89]***** | | **1.54 [1.27-1.87]***** | | | 1.18 [0.95-1.46] | | |  | | 1.18 [0.95-1.47] | | |  |
| Refused/DK | 27.6 | 0.97 [0.74-1.27] |  |  | 0.99 [0.75-1.31] |  | | 1.00 [0.75-1.33] |  | | 0.95 [0.69-1.31] | | |  | | 0.99 [0.71-1.37] | | |
| Important people's thoughts on your NVP use |  |  |  |  |  |  | |  |  | |  | | |  | |  | | |
| Most/All approve | 35.1 | 0.89 [0.66-1.19] |  |  | 0.91 [0.67-1.24] |  | | 0.90 [0.66-1.23] |  | | 0.84 [0.59-1.18] | | |  | | 0.75 [0.53-1.07] | | |
| Half approve & half disapprove | 38.4 | *ref* |  |  | *ref* |  | | *ref* |  | | *ref* | | |  | | *ref* | | |
| Most/All disapprove | 47.7 | **1.31 [1.02-1.67]*** | |  | 1.18 [0.90-1.54] |  | | 1.18 [0.91-1.55] |  | | 1.18 [0.88-1.58] | | |  | | 1.24 [0.92-1.68] | | |
| Refused/DK/NHV | 34.7 | 0.90 [0.73-1.11] |  |  | 0.92 [0.73-1.16] |  | | 0.93 [0.74-1.18] |  | | 0.97 [0.74-1.27] | | |  | | 1.03 [0.78-1.35] | | |
| **Descriptive interpersonal norms** | | |  |  |  |  | |  |  | |  | | |  | |  | | |
| Number of 5 closest  friends smoking |  |  |  |  |  |  | |  |  | |  | | |  | |  | | |
| 0 | 41.8 | *ref* |  |  | NA |  | | *ref* |  | | *ref* | | |  | | *ref* | | |
| 1-5 | 38.0 | **0.81 [0.68-0.97]*** | |  | NA |  | | 0.89 [0.74-1.07] |  | | 0.92 [0.75-1.13] | | |  | | 0.92 [0.75-1.14] | | |
| Refused/DK/NRF | 33.5 | **0.73 [0.58-0.91]**** | |  | NA |  | | 1.37 [0.85-2.19] |  | | 1.64 [0.98-2.73] | | |  | | 1.67 [0.99-2.81] | | |
| Number of 5 closest  friends using NVPs |  |  |  |  |  |  | |  |  | |  | | |  | |  | | |
| 0 | 39.2 | *ref* |  |  | NA |  | | *ref* |  | | *ref* | | |  | | *ref* | | |
| 1-5 | 43.7 | **1.37 [1.04-1.79]*** | |  | NA |  | | **1.39 [1.05-1.84]*** | | | **1.44 [1.05-1.97]*** | | | | | **1.40 [1.02-1.91]*** | | |
| Refused/DK/NRF | 32.9 | **0.78 [0.65-0.94]**** | |  | NA |  | | **0.60 [0.38-0.93]*** | | | **0.56 [0.35-0.90]*** | | | | | **0.55 [0.33-0.89]*** | | |
| **Baseline quit intention** | |  |  |  |  |  | |  |  | |  | | |  | |  | | |
| No plans | 14.4 | NA |  |  | NA |  | | NA |  | | *ref* | | |  | | *ref* | | |
| Beyond 6 months | 35.0 | NA |  |  | NA |  | | NA |  | | **2.90 [2.27-3.70]***** | | |  | | **2.77 [2.16-3.55]***** | | |
| Next 6 months | 65.0 | NA |  |  | NA |  | | NA |  | | **9.31 [7.09-12.21]***** | | | | | **9.57 [7.25-12.62]***** | | |
| Next month | 78.6 | NA |  |  | NA |  | | NA |  | | **15.76 [11.03-22.51]***** | | | | | **16.03 [11.20-22.96]***** | | |
| Refused/DK | 28.9 | NA |  |  | NA |  | | NA |  | | **2.08 [1.56-2.76]***** | | |  | | **2.05 [1.53-2.74]***** | | |
| **NVP uptake by follow-up**  No  Yes | 33.9  53.2 | NA  NA |  |  | NA  NA |  | | NA  NA |  | | NA  NA | | |  | | *Ref*  **2.51 [2.02-3.12]***** | | |

*Note.* n=number of unique individuals; N=number of observations; AOR=adjusted odds ratio; CI=confidence interval; *ref*=reference group; NHV=never heard of NVP use; NRF=no regular friends; NA=not applicable;

Model 1= individual analysis of social norm measures for smoking and NVP use plus covariates such as country, gender, ethnicity, age, education, income, smoking status, wave of recruitment, and survey wave;

Model 2= concurrent analysis of norm measures after adding in injunctive norm measures for smoking and NVP use plus covariates such as country, gender, ethnicity, age, education, income, smoking status, wave of recruitment, and survey wave;

Model 3= concurrent analysis of norm measures after adding in descriptive norm measures for smoking and NVP use plus covariates such as country, gender, ethnicity, age, education, income, smoking status, wave of recruitment, and survey wave;

Model 4= concurrent analysis of full set of social norm measures for smoking and NVP use after adding in quit intention plus covariates such as country, gender, ethnicity, age, education, income, smoking status, quit intention, wave of recruitment, and survey wave;

Model 5= concurrent analysis of full set of social norm measures for smoking and NVP use after adding in NVP uptake by follow-up plus covariates such as country, gender, ethnicity, age, education, income, smoking status, quit intention, wave of recruitment, and survey wave;
